# Supplementary material for: Epistatic interactions between the high pathogenicity island and other iron uptake systems shape Escherichia coli extra-intestinal virulence
Source: Nat Commun. 2023 Jun 20;14:3667. doi: 10.1038/s41467-023-39428-y (PMC10282060; doi:10.1038/s41467-023-39428-y)
Supplement: Supplementary file 1 — Supplementary information [file 41467_2023_39428_MOESM1_ESM.pdf]

Supplementary Information

Epistatic interactions between the high pathogenicity island and other iron uptake systems shape *Escherichia coli* extra-intestinal virulence

Guilhem Royer[1,2,3,4,5], Olivier Clermont[1], Julie Marin[1,6], Bénédicte Condamine[1], Sara Dion[1], François Blanquart[7], Marco Galardini[8,9], Erick Denamur[1,10]

1. Université Paris Cité, IAME, INSERM, Paris, France
2. Département de Prévention, Diagnostic et Traitement des Infections, Hôpital Henri Mondor, Créteil, France
3. LABGeM, Génomique Métabolique, Genoscope, Institut François Jacob, CEA, CNRS, Université Paris-Saclay, Evry, France
4. EERA Unit "Ecology and Evolution of Antibiotics Resistance," Institut Pasteur-Assistance Publique/Hôpitaux de Paris-Université Paris-Saclay, Paris, France
5. UMR CNRS 3525, Paris, France
6. Université Sorbonne Paris Nord, IAME, INSERM, Bobigny, France
7. Center for Interdisciplinary Research in Biology, CNRS, Collège de France, PSL Research University, Paris, France
8. Institute for Molecular Bacteriology, TWINCORE Centre for Experimental and Clinical Infection Research, a joint venture between the Hannover Medical School (MHH) and the Helmholtz Centre for Infection Research (HZI), Hannover, Germany
9. Cluster of Excellence RESIST (EXC 2155), Hannover Medical School (MHH), Hannover, Germany
10. AP-HP, Hôpital Bichat, Laboratoire de Génétique Moléculaire, Paris, France

Supplementary Figures

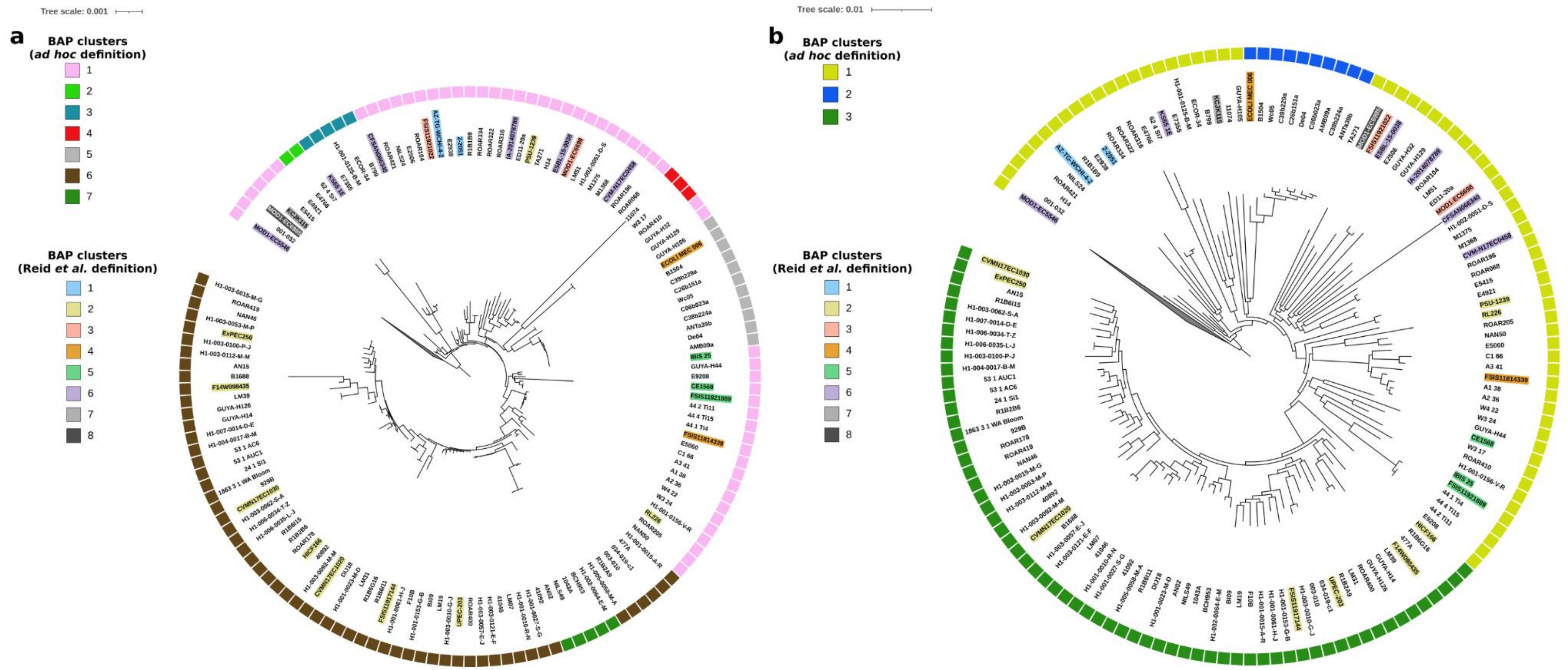

Figure S1: Comparison of BAP clustering according to the alignment used to compute the phylogenetic tree. **a** Phylogenetic tree of the STc58 computed from the alignment of the core genes as defined by Roary. The outermost circle represents the BAP clusters computed from this alignment using the optimized bap model. The genomes from the study by Reid *et al.*[1] are background colored according to the BAP clusters previously defined by Reid *et al.*[1] The tree is rooted on the ST155 strains. **b** Phylogenetic tree the STc58 computed from the recombination-free coregenome alignment. The outermost circle represents the BAP clusters computed from this alignment using the optimized bap model. The genomes from the study by Reid *et al.*[1] are background colored according to the BAP clusters previously defined by Reid *et al.*[1] The tree is rooted on the ST155 strains.

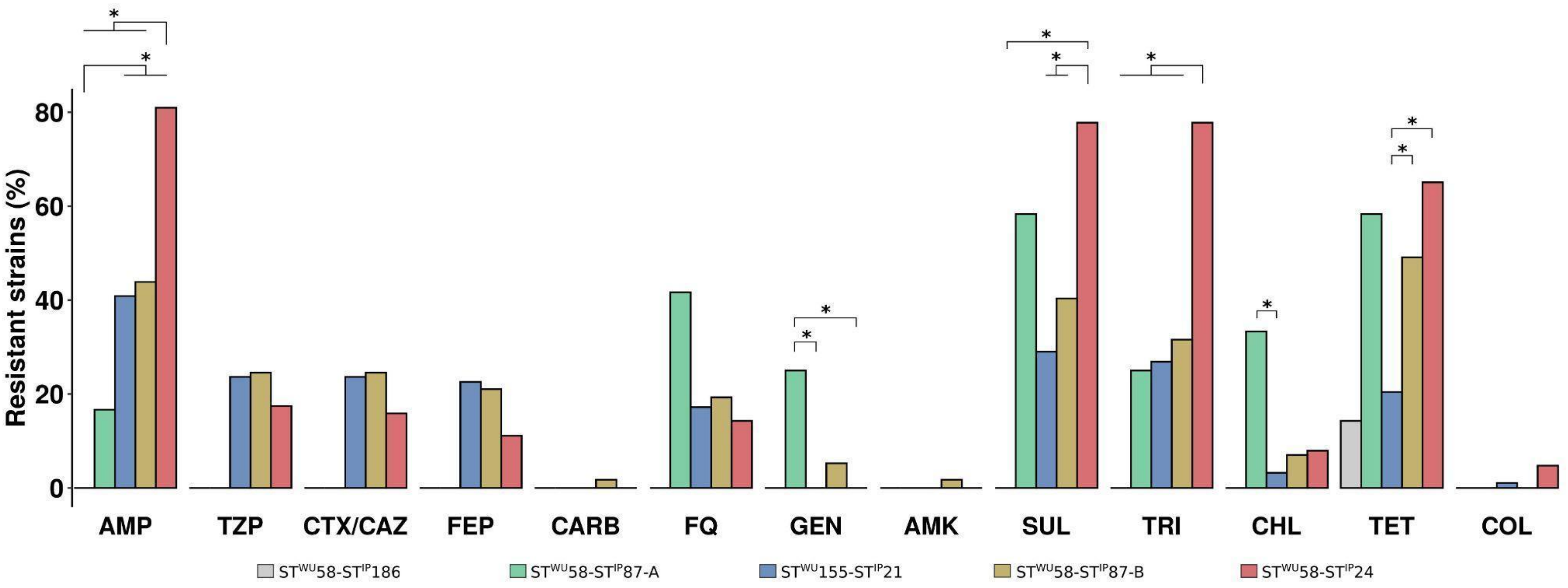

Figure S2: Predicted resistance phenotypes of the strains (Two-sided Fisher exact test with Bonferroni adjusted p-value). The results are presented as the percentage of resistant strains for thirteen antibiotics of clinical and/or veterinary importance. Bars are coloured according to the CC87 subgroups. We predicted the phenotypes from the presence of genes or mutations in genomes as in Royer *et al.*[2] and based on the genotype to phenotype predictions described in Supplementary Data 3. Significant differences are highlighted by asterisks. Exact p-values are available in Supplementary Data 2.

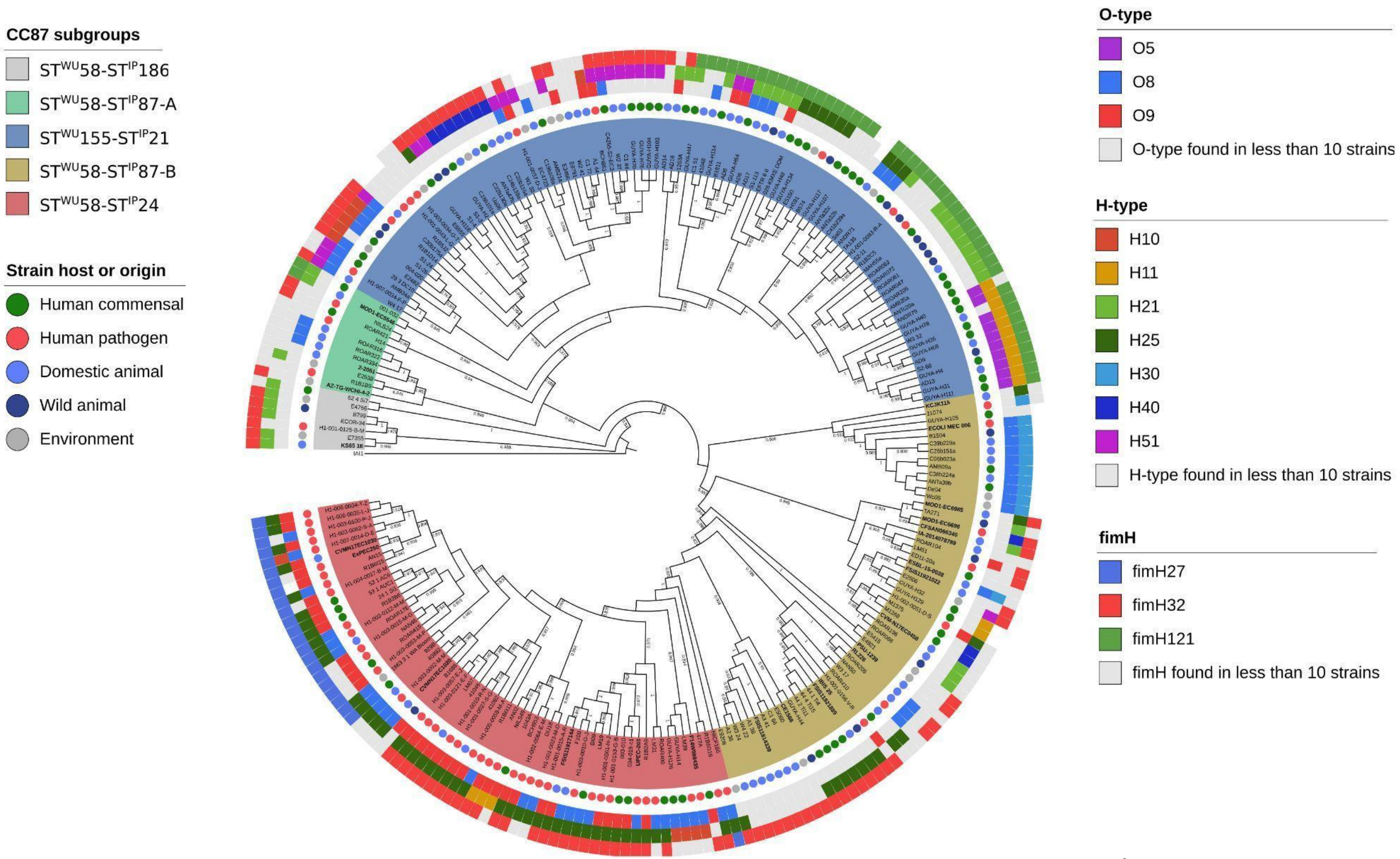

Figure S3: Maximum likelihood core genome phylogenetic tree of the 232 B1 phylogroup CC87 (Institut Pasteur scheme numbering)[3] strains. The five CC87 subgroups ($ST^{WU}58$-$ST^{IP}186$, $ST^{WU}58$-$ST^{IP}87$-A, $ST^{WU}155$-$ST^{IP}21$, $ST^{WU}58$-$ST^{IP}87$-B, $ST^{WU}58$-$ST^{IP}24$) based on the Warwick University[4] and Institut Pasteur[3] MLST schemes are highlighted in color. The host or origin of the strain is highlighted by coloured circles. O-types (*wzm, wzt, wzx* or *wzy*), H-types and fimH-types are represented by coloured rectangles from the inside to the outside. Only antigens found in more than 10 strains are coloured, the others are in grey. The 26 ST58 genomes obtained from the study by Reid et al. are in bold. For the sake of readability, branch lengths are ignored and local support values higher than 0.7 are shown.

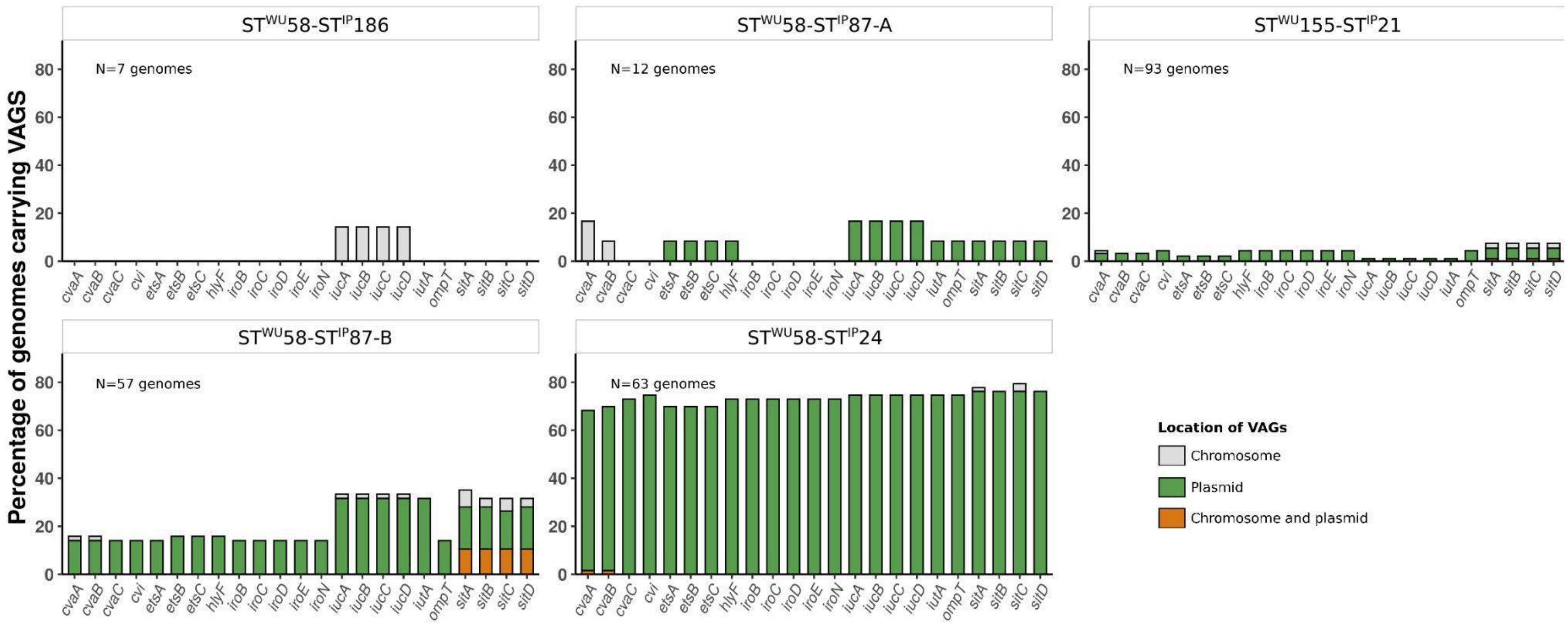

Figure S4: Distribution of VAGs and predicted location among the 232 B1 phylogroup CC87 genomes. Results are presented as the percentage of genomes carrying VAGs among each CC87 subgroup depending on their predicted location. Plasmidic location is highlighted in green, chromosomal location in grey and cases with both chromosomal and plasmidic location in orange.

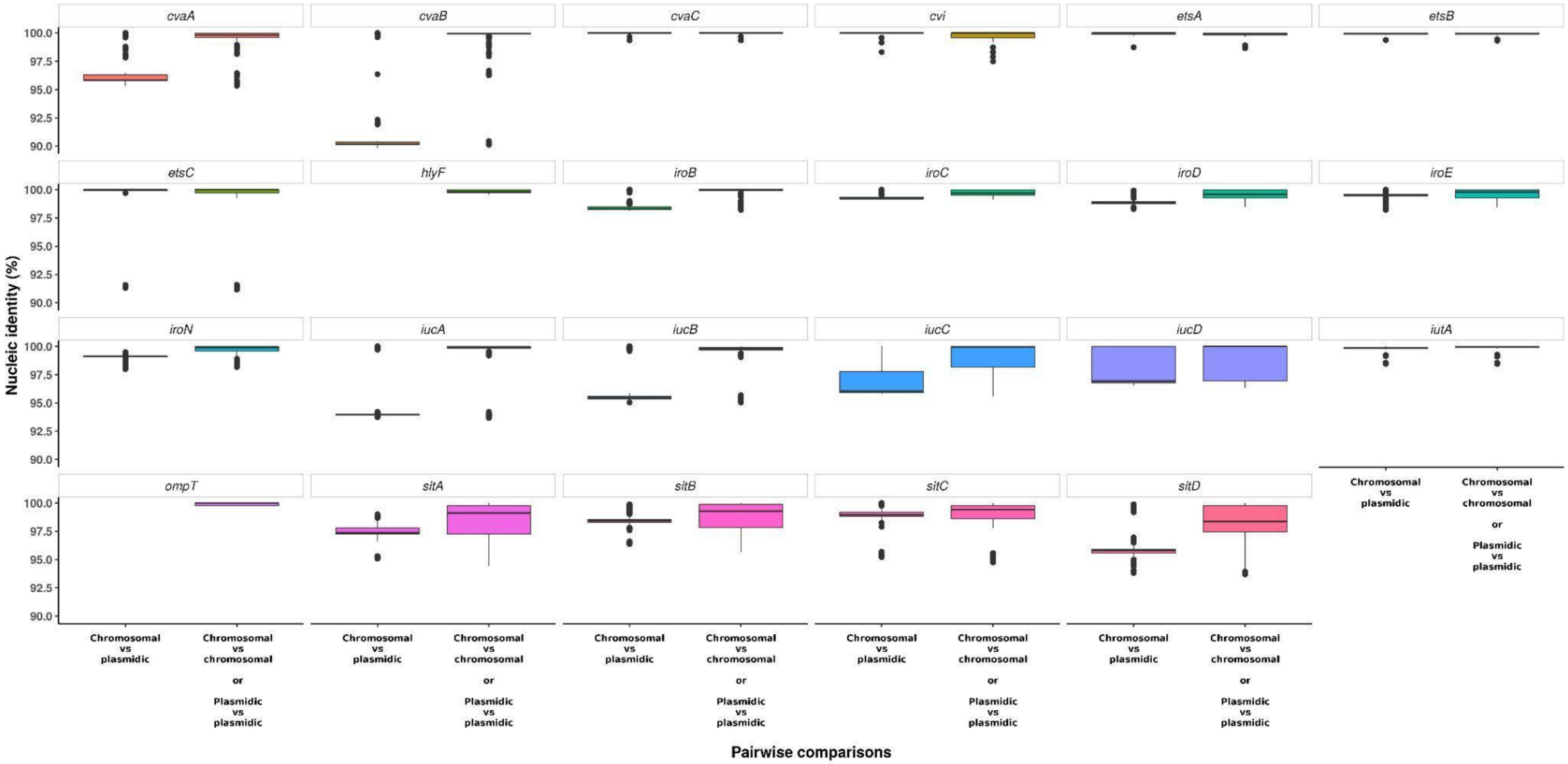

Figure S5: Pairwise comparisons of VAG sequences according to their predicted location (n=370 biologically independent *Escherichia* genomes). We ran an all-against-all blastN analysis among the 370 genomes of *Escherichia*. The nucleic identity distributions of all pairwise comparisons are plotted as boxplots against the predicted location of VAGs within a given pair. The upper and lower limits of box-plots represent 75th and 25th quartile, the centre line represents the median and the whiskers extend to 1.5 × IQR. Dots represent values outside these ranges.

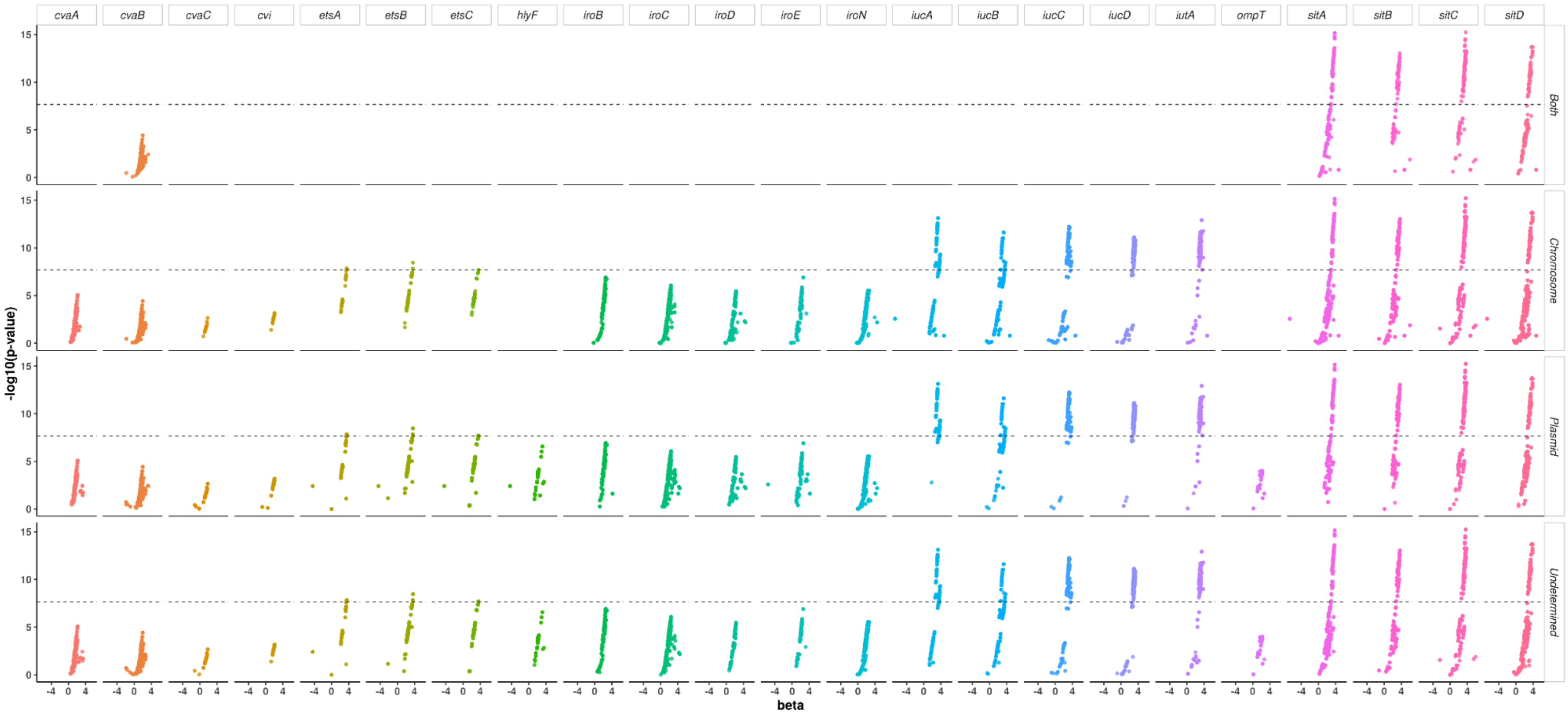

Figure S6: Association between virulence and unitigs belonging to the ColV plasmid VAGs at the genus level according to their predicted location (likelihood ratio test). The GWAS results were obtained from a previous study performed on 370 strains of *Escherichia*[5]. The p-value of the association is shown on the y-axis, the effect size (beta) on the x-axis and the significance level of the GWAS analysis with a dotted line (Bonferroni multiple-testing corrected p-value). Each facet represents the result for a given VAG in a given predicted location (i.e. chromosomal, plasmidic, both or undetermined).

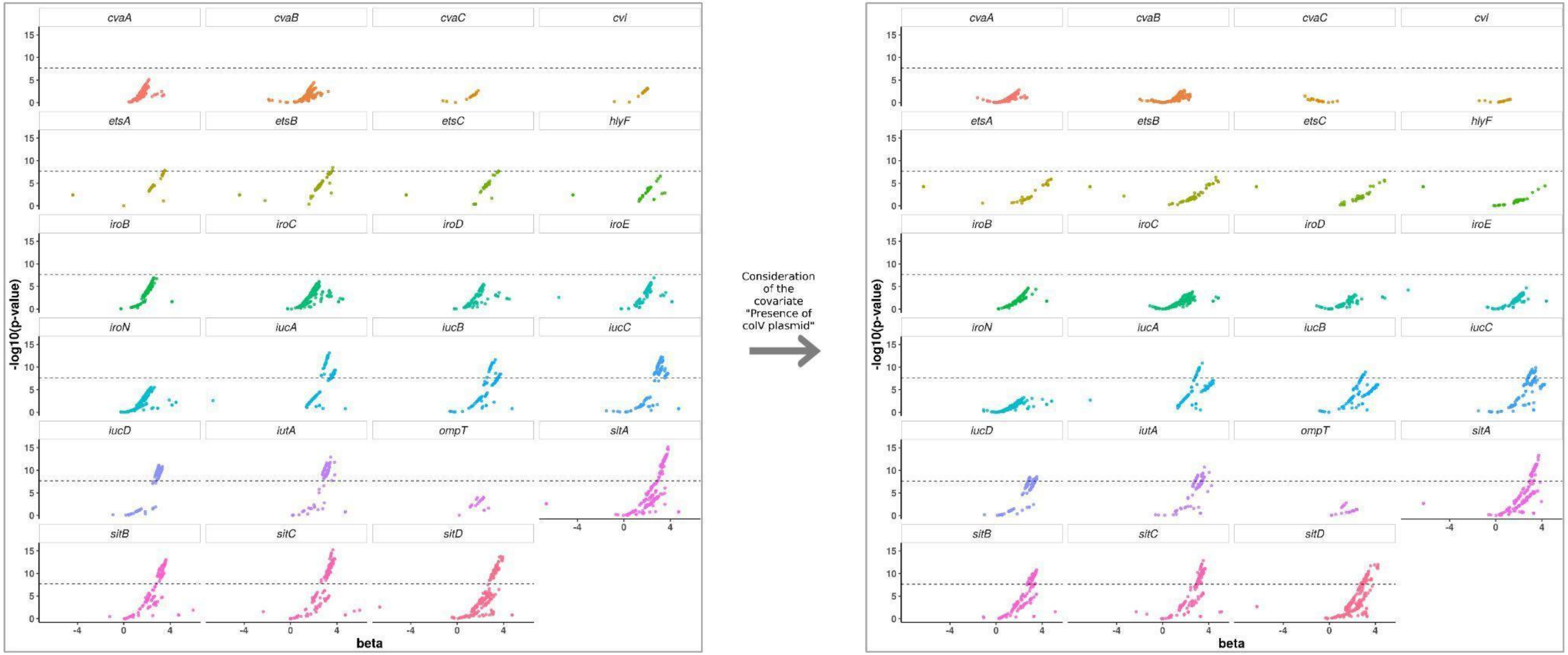

Figure S7: Association between the unitigs within each VAG used to infer the presence of the ColV plasmid and virulence in mice after considering ColV plasmids as covariate (likelihood ratio test). The scatter plots on the left hand side of the figure represent the results for the 370 strains of the genus *Escherichia*[5] without taking into account the presence of the ColV plasmid. The scatter plots on the right hand side of the figure represent the results of the same analysis but considering the presence of the ColV plasmid as a covariate during the GWAS analysis. The p-value of the association is shown on the y-axis, the effect size (beta) on the x-axis and the significance level of the GWAS analysis with a dotted line (Bonferroni multiple-testing corrected p-value).

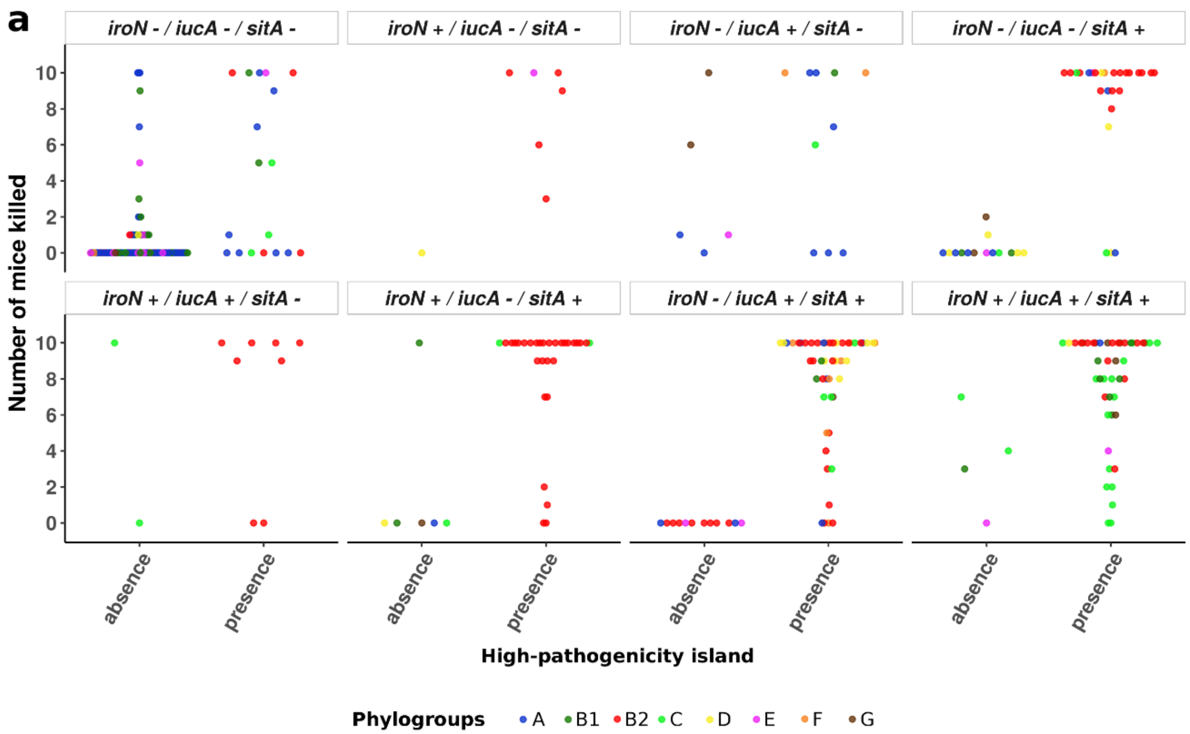

b

Odds ratios for the status “killer” (at least 9 mice killed) as a function of the presence of the HPI

VAG combinations	Odds ratio (95% CI)	p-value
iroN - / iucA - / sitA -	10.36 (2.12 - 57.65)	1.33E-03
iroN + / iucA - / sitA -	infinite (0.3 - infinite)	4.29E-01
iroN - / iucA + / sitA -	3.66 (0.24-235.26)	5.80E-01
iroN - / iucA - / sitA +	infinite (9.0255 - infinite)	5.01E-07
iroN + / iucA + / sitA -	2.65 (0.03 - 273.20)	1.00E+00
iroN + / iucA - / sitA +	17.91 (1.61 - 976.90)	6.17E-03
iroN - / iucA + / sitA +	infinite (4.05 - infinite)	4.97E-05
iroN + / iucA + / sitA +	infinite (0.73 - infinite)	5.02E-02

Figure S8: Virulence according to VAG combinations, HPI and phylogroups. **a** Number of mice killed over ten according to the combination of iron acquisition-related VAGs *iroN*, *iucA*, *sitA*. In each facet, each point represents the number of mice killed by a given strain according to the combination of VAGs it carries. Points are colored according to the phylogroup belonging of the strains. **b** Odds ratio (95% confidence interval) and p-value for the status “killer” (i.e. at least 9/10 mice killed)[6] (Two-sided Fisher exact test) as a function of the presence of the HPI in strains carrying different combinations of VAGs.

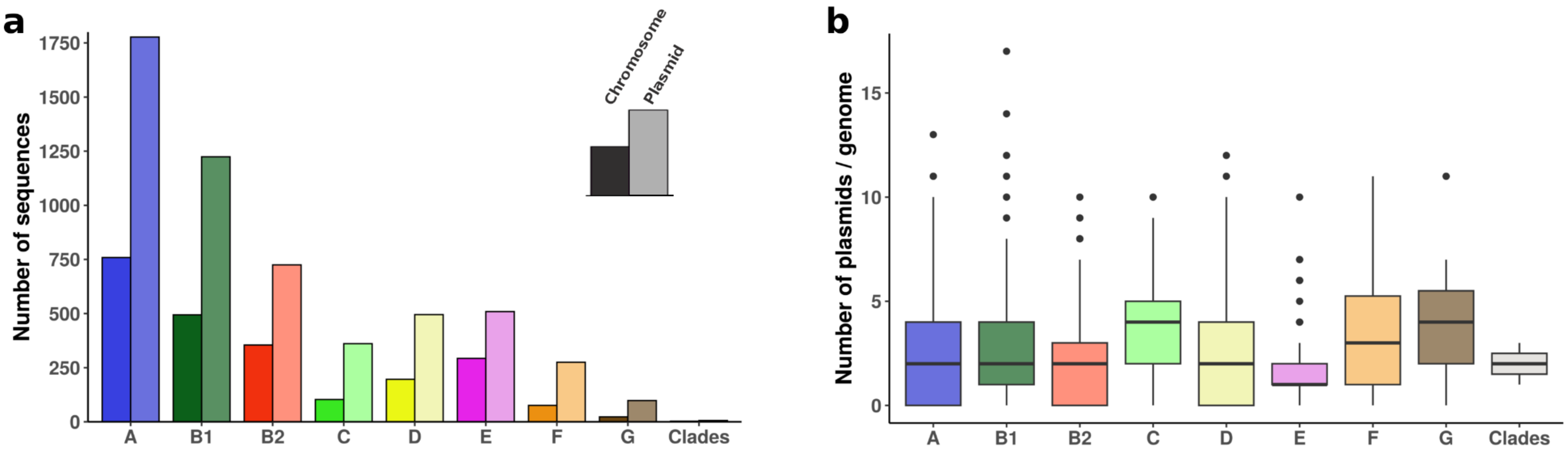

c

		PlaScope prediction		
		Chromosome	Plasmid	Unclassified
Refseq classification	Chromosome	2301	1	0
	Plasmid	235	5154	81

Figure S9: Assessment of PlaScope[7] performances on the 2302 genomes of *E. coli* from RefSeq. **a** Total number of chromosomal and plasmidic sequences according to the phylogroup among the RefSeq dataset. **b** Distribution of the number of plasmid sequences according to the phylogroup among the RefSeq dataset (n=2302 biologically independent *E. coli* genomes). The upper and lower limits of box-plots represent 75th and 25th quartile, the centre line represents the median and the whiskers extend to 1.5 × IQR. Dots represent values outside these ranges. **c** Comparison of PlaScope and RefSeq classifications. From these results it appears that PlaScope identifies chromosome and plasmid sequences with both high recall (0.94) and specificity (>0.99).

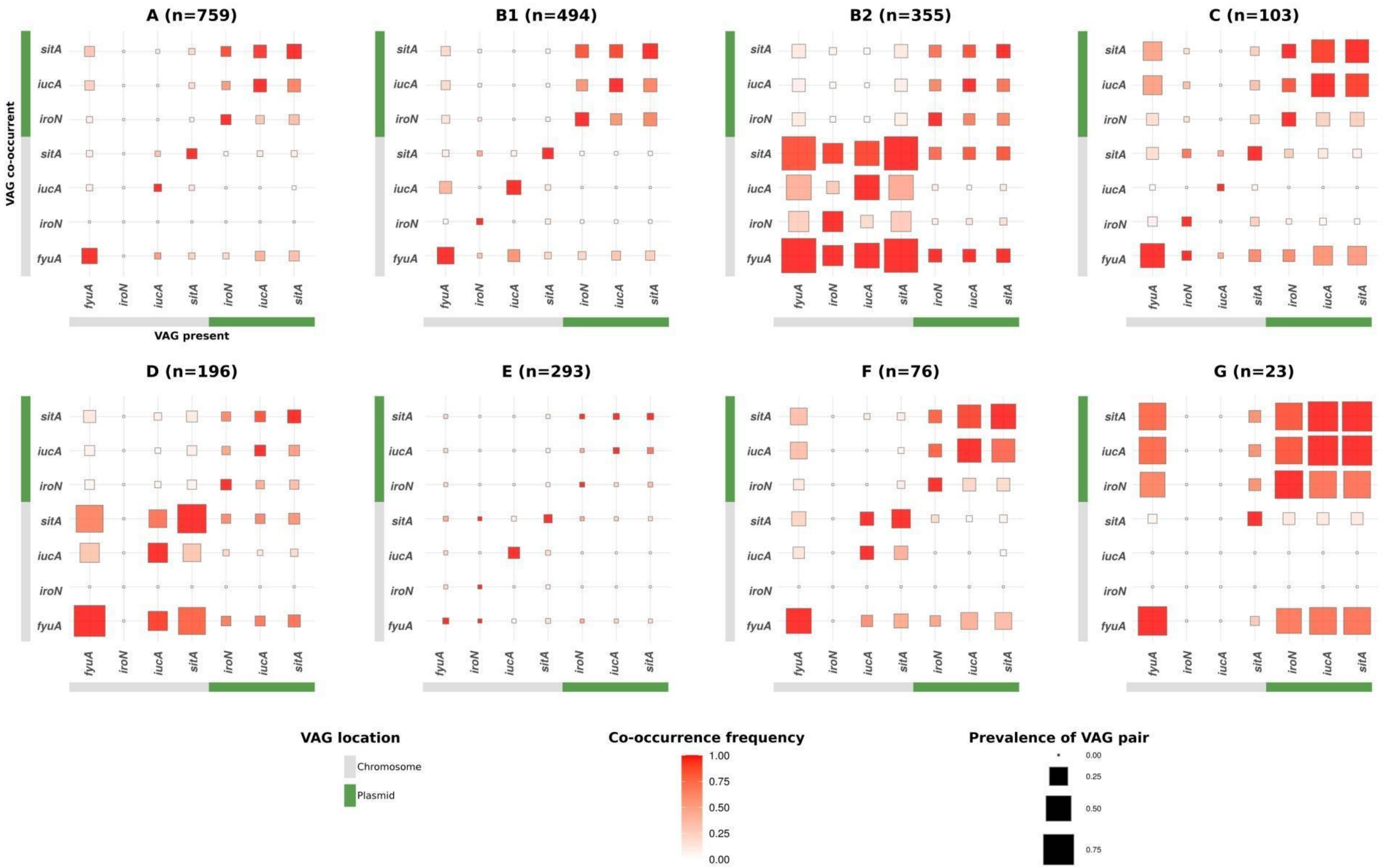

Figure S10: Co-occurrence frequency and prevalence of iron acquisition-related VAG pairs among 2299 fully circularized genomes of *E. coli* belonging to phylogroups A, B1, B2, C, D, E, F, G. For each given VAG on the x-axis, the frequency of co-occurrence with the VAGs on the y-axis is highlighted by a colour gradient. The size of each square is proportional to the prevalence of the VAG pair in the given ST/STc. VAGs are separated according to their location on the chromosome in grey or on the plasmid in green. *Escherichia* Clade I genomes are not included due to their very low prevalence in the data set (n=3). Odds ratio to test for associations between chromosomal and/or plasmidic VAGs in a given phylogroup are available in Supplementary Data 4.

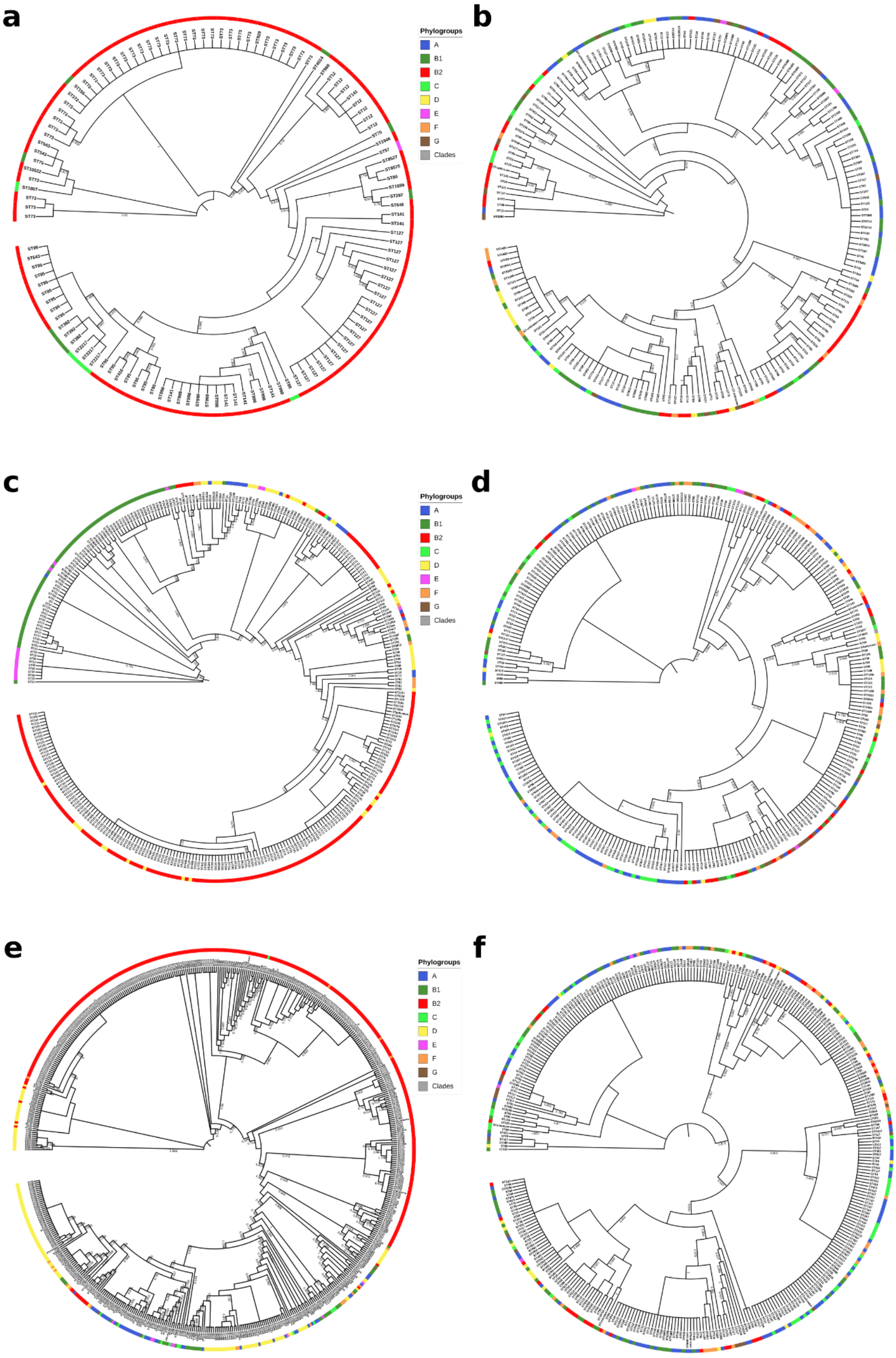

Figure S11: Phylogenetic trees of the operon *iro*, *iuc* and *sit*. **a** Phylogenetic tree computed from the alignment of chromosomal *iro* operon. **b** Phylogenetic tree computed from the alignment of plasmidic *iro* operon. **c**

Phylogenetic tree computed from the alignment of chromosomal *iuc* operon. **d** Phylogenetic tree computed from the alignment of plasmidic *iuc* operon. **e** Phylogenetic tree computed from the alignment of chromosomal *sit* operon. **f** Phylogenetic tree computed from the alignment of plasmidic *sit* operon. The phylogroup of the strains are highlighted in color in the outermost circles and MLST of the strain is used as leaf label. For the sake of readability, branch lengths are ignored and local support values higher than 0.7 are shown.

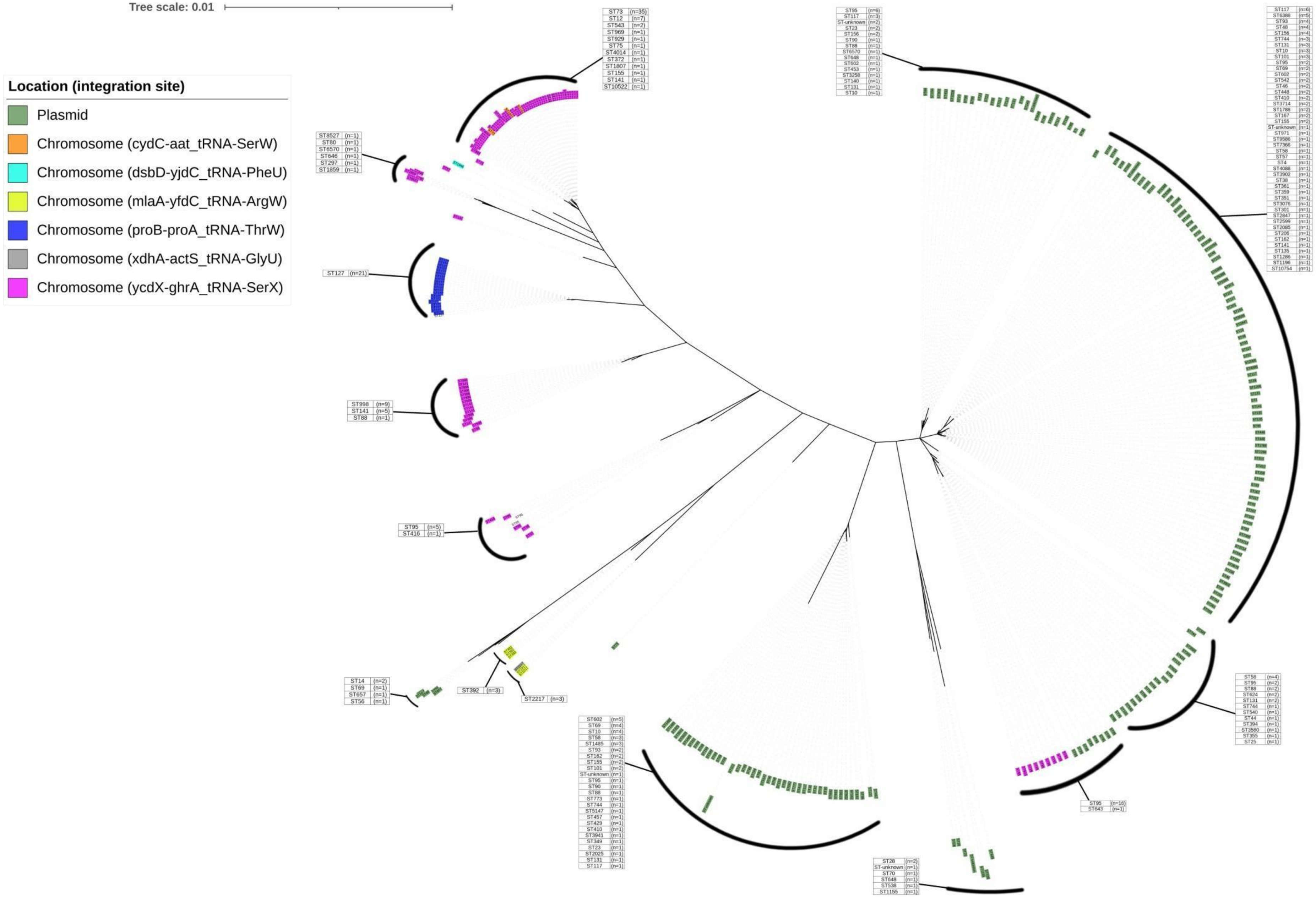

Figure S12. Unrooted phylogenetic tree of the operon *iro* including both plasmid and chromosomal sequences. The location and the integration sites in the case of chromosomal location are highlighted in color. For each cluster of sequences in the tree, the associated table shows the STs of the strains carrying the operon and the number of these STs in the cluster.

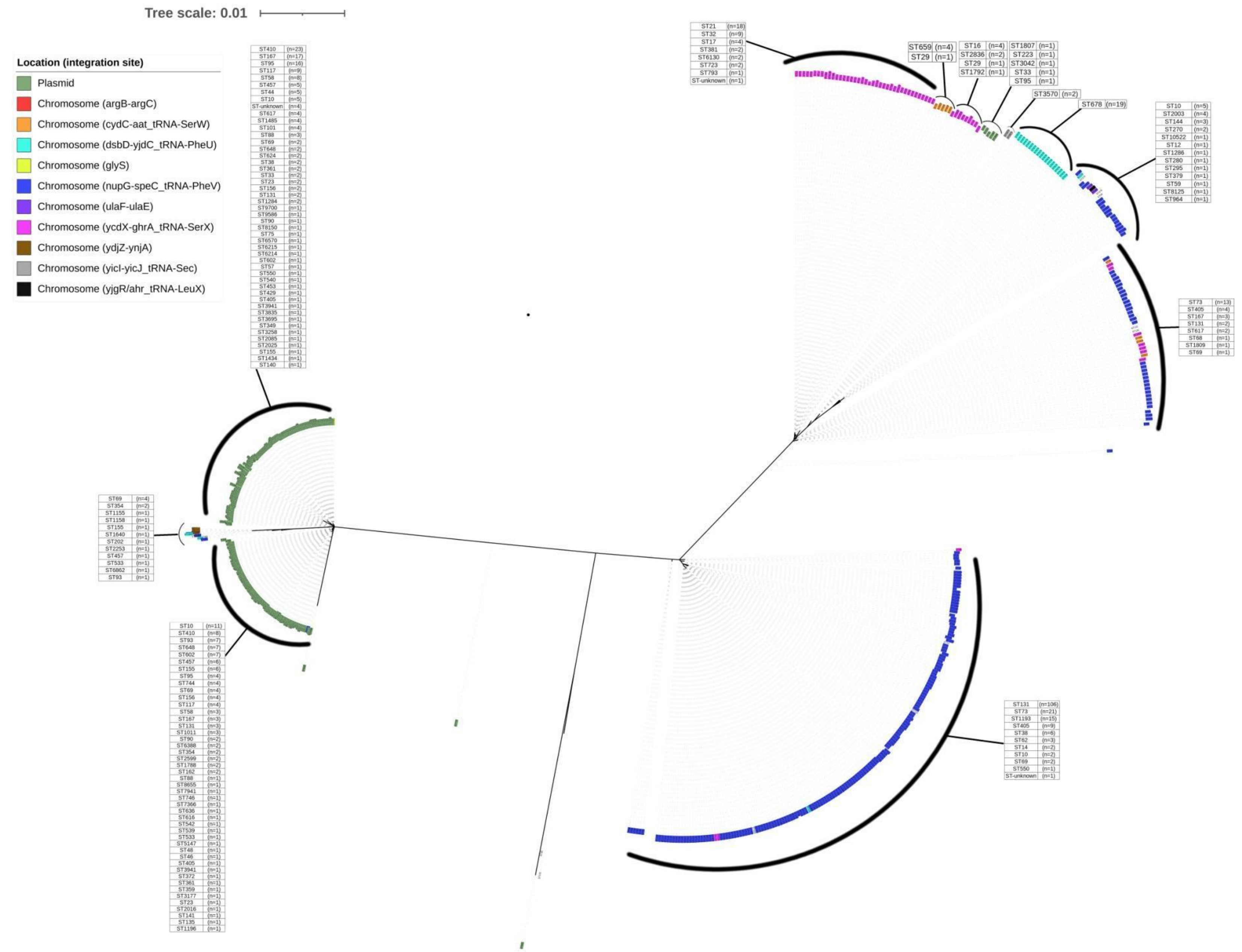

Figure S13. Unrooted phylogenetic tree of the operon *aer* including both plasmid and chromosomal sequences. The location and the integration sites in the case of chromosomal location are highlighted in color. For each cluster of sequences in the tree, the associated table shows the STs of the strains carrying the operon and the number of these STs in the cluster.

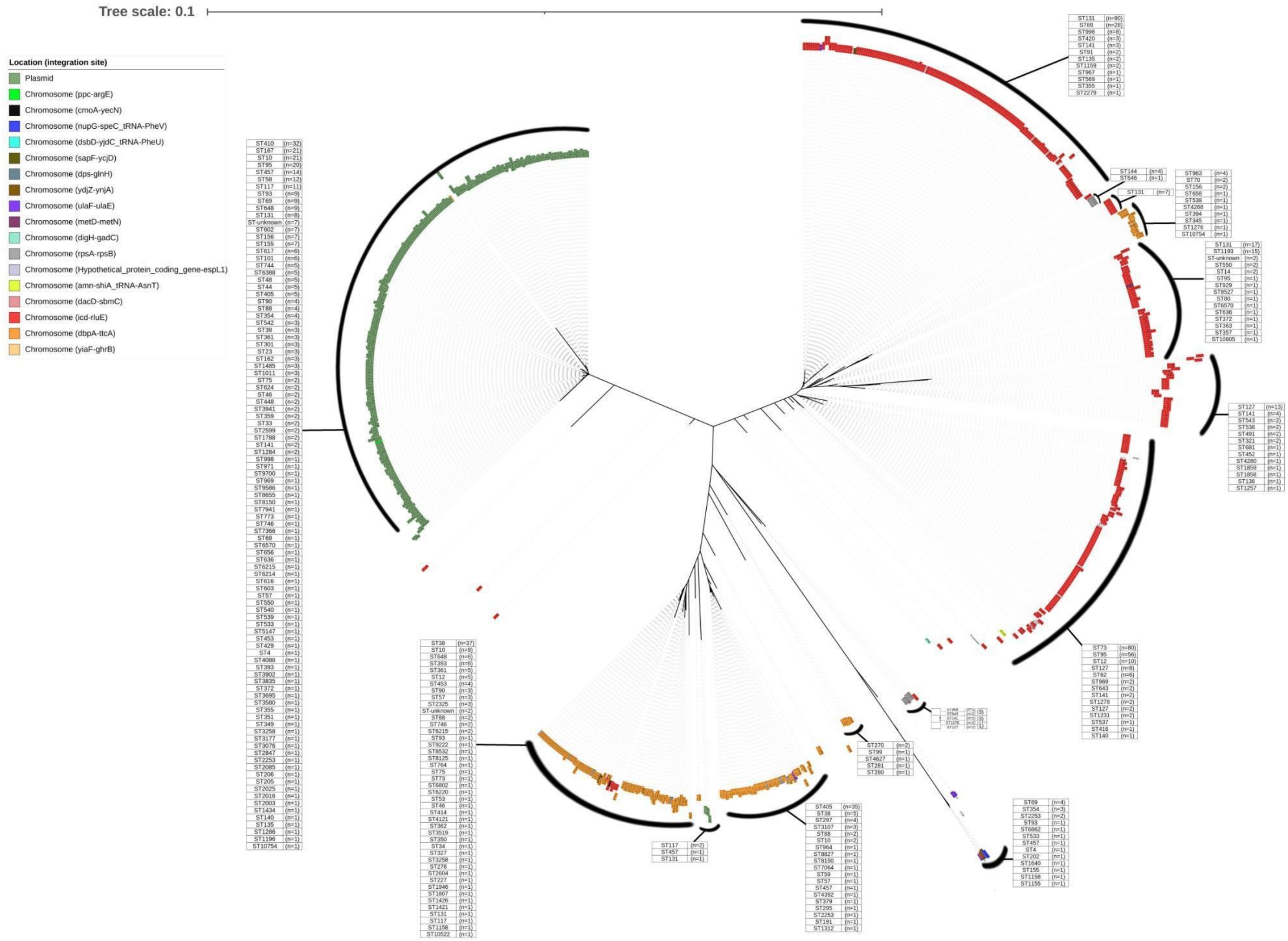

Figure S14. Unrooted phylogenetic tree of the operon *sit* including both plasmid and chromosomal sequences. The location and the integration sites in the case of chromosomal location are highlighted in color. For each cluster of sequences in the tree, the associated table shows the STs of the strains carrying the operon and the number of these STs in the cluster.

Supplementary references

1. Reid, C. J. *et al.* A role for ColV plasmids in the evolution of pathogenic *Escherichia coli* ST58. *Nat. Commun.* **13**, 683 (2022).

2. Royer, G. *et al.* Phylogroup stability contrasts with high within sequence type complex dynamics of *Escherichia coli* bloodstream infection isolates over a 12-year period. *Genome Med.* **13**, 77 (2021).

3. Jaureguy, F. *et al.* Phylogenetic and genomic diversity of human bacteremic *Escherichia coli* strains. *BMC Genomics* **9**, 560 (2008).

4. Wirth, T. *et al.* Sex and virulence in *Escherichia coli*: an evolutionary perspective. *Mol. Microbiol.* **60**, 1136–1151 (2006).

5. Galardini, M. *et al.* Major role of iron uptake systems in the intrinsic extra-intestinal virulence of the genus *Escherichia* revealed by a genome-wide association study. *PLoS Genet.* **16**, e1009065 (2020).

6. Johnson, J. R. *et al.* Experimental mouse lethality of *Escherichia coli* isolates, in relation to accessory traits, phylogenetic group, and ecological source. *J. Infect. Dis.* **194**, 1141–1150 (2006).

7. Royer, G. *et al.* PlaScope: a targeted approach to assess the plasmidome from genome assemblies at the species level. *Microb. Genomics* **4**, (2018).
