# Supplementary material for: Epistatic interactions between the high pathogenicity island and other iron uptake systems shape Escherichia coli extra-intestinal virulence
Source: Nat Commun. 2023 Jun 20;14:3667. doi: 10.1038/s41467-023-39428-y (PMC10282060; doi:10.1038/s41467-023-39428-y)
Supplement: Supplementary file 3 — Description of Additional Supplementary Files [file 41467_2023_39428_MOESM3_ESM.pdf]

Description of Additional Supplementary Data:

Supplementary data 1. Principal characteristics of the 232 Escherichia coli CC87 strains studied

Supplementary data 2. KruskalWallis test p-values after Bonferroni correction of VAGs, two-sided Fisher exact test p-values after Bonferroni correction of antibiotic resistance data.

Supplementary data 3. Antibiotic resistance prediction according to genes/mutations.

Supplementary data 4. Odds ratio for the cooccurrences of VAGs fyuA, iroN, iucA and sitA in the different ST/STc and phylogroups (Two-sided Fisher exact test).

Supplementary data 5. Chromosomal insertion sites of iroN.

Supplementary data 6. Chromosomal insertion sites of iucA.

Supplementary data 7. Chromosomal insertion sites of sitA.

Supplementary data 8. Associations between the presence of HPI and the presence of another iron-capture system using Pagel's model. The p-values were corrected using the Bonferroni correction method. Significant results are in bold.

Supplementary data 9. Recapitulative table of molecular defaults (nonsense, small and large deletion and insertion) found on the four iron capture systems and on core mlst genes. We computed the inactivaton rate as the number of strains with at least one defaults over gene length.

Supplementary data 10. Associations between the inactivation of genes in one ironcapture system and the presence of another iron-capture system using Pagel's model. The p-values were corrected using the Bonferroni correction method. Significant results are in bold.

Supplementary data 11. List of the 2302 fully sequenced genomes from RefSeq.
