# Supplementary material for: Epistatic interactions between the high pathogenicity island and other iron uptake systems shape Escherichia coli extra-intestinal virulence
Source: Nat Commun. 2023 Jun 20;14:3667. doi: 10.1038/s41467-023-39428-y (PMC10282060; doi:10.1038/s41467-023-39428-y)
Supplement: Supplementary file 15 — Reporting Summary [file 41467_2023_39428_MOESM15_ESM.pdf]

nature portfolio

Corresponding author(s): Erick Denamur

Last updated by author(s): Jun 2, 2023

Reporting Summary

Nature Portfolio wishes to improve the reproducibility of the work that we publish. This form provides structure for consistency and transparency in reporting. For further information on Nature Portfolio policies, see our Editorial Policies and the Editorial Policy Checklist.

Statistics

For all statistical analyses, confirm that the following items are present in the figure legend, table legend, main text, or Methods section.

n/a	Confirmed	
[ ]	[x]	The exact sample size (*n*) for each experimental group/condition, given as a discrete number and unit of measurement
[ ]	[x]	A statement on whether measurements were taken from distinct samples or whether the same sample was measured repeatedly
[ ]	[x]	The statistical test(s) used AND whether they are one- or two-sided<br>*Only common tests should be described solely by name; describe more complex techniques in the Methods section.*
[ ]	[x]	A description of all covariates tested
[ ]	[x]	A description of any assumptions or corrections, such as tests of normality and adjustment for multiple comparisons
[ ]	[x]	A full description of the statistical parameters including central tendency (e.g. means) or other basic estimates (e.g. regression coefficient) AND variation (e.g. standard deviation) or associated estimates of uncertainty (e.g. confidence intervals)
[ ]	[x]	For null hypothesis testing, the test statistic (e.g. *F*, *t*, *r*) with confidence intervals, effect sizes, degrees of freedom and *P* value noted<br>*Give P values as exact values whenever suitable.*
[x]	[ ]	For Bayesian analysis, information on the choice of priors and Markov chain Monte Carlo settings
[x]	[ ]	For hierarchical and complex designs, identification of the appropriate level for tests and full reporting of outcomes
[x]	[ ]	Estimates of effect sizes (e.g. Cohen's *d*, Pearson's *r*), indicating how they were calculated

Our web collection on statistics for biologists contains articles on many of the points above.

Software and code

Policy information about availability of computer code

Data collection	ncbi-genome-download v0.3.1 (https://github.com/kblin/ncbi-genome-download) was used to retrieve complete genome of E. coli.
Data analysis	mlst v2.19.0 (https://github.com/tseemann/mlst) was used to determine sequence type of the strains based on Warwick University and Pasteur Institute schemes.<br>Abricate v0.7 was used to search for serotype and fimH based on ecoh, serotypefinder and fimtyper databases. It was also used to search for virulence associated genes and antibiotic resistance genes based on VirulenceFinder, VFDB, a custom database of virulence genes and Resfinder.<br>PlaScope v1.3 was used to classify contigs as chromosomal or plasmidic based on an E. coli database available on Zenodo (10.5281/zenodo.1311641).<br>Snippy v4.4.0 was used to generate a core-genome alignement of CC87 genomes.<br>Gubbins v2.3.4 was used to filter recombination from the core-genome alignment.<br>FastTree v2.1.11 was used to compute phylogenetic trees.<br>Pyseer v1.3.9 was used to perform a GWAS analysis based on unitigs computed with unitig-caller v1.2.1 and gene presence/absence determined using Roary v3.12.0.<br>Prokka v1.14.5 was used to annotate genomes were required.<br>bwa 0.7.17 and bedtols 2.30.0 were used to map back significant unitigs to reference genomes.<br>R package "ggplot2" was used to draw plots.<br>Clinker v0.0.23 was used to generate physical maps of genomes.<br>Blast v2.7.1 was used to compare virulence associated gene sequences.<br>Ppanggolin v1.2.74 was used to compute a pangenome of complete genomes of E. coli. This version also includes PanRGP that was used to

analyse the region of genome plasticity.
Mafft v7.31.0 was used to generate multiple alignment of some virulence associated genes.
R package "ape" v5.1 was used to compute patristic distances from the phylogenetic tree of the virulence associated phylogenetic tree.
R package "phytools" was used to correct for phylogenetic structure using Pagel's model when searching for associations between VAGs at the whole species level and associations between the inactivation of a given VAG and the presence/absence of other VAGs.

For manuscripts utilizing custom algorithms or software that are central to the research but not yet described in published literature, software must be made available to editors and reviewers. We strongly encourage code deposition in a community repository (e.g. GitHub). See the Nature Portfolio guidelines for submitting code & software for further information.

Data

Policy information about availability of data

All manuscripts must include a data availability statement. This statement should provide the following information, where applicable:

- Accession codes, unique identifiers, or web links for publicly available datasets
- A description of any restrictions on data availability
- For clinical datasets or third party data, please ensure that the statement adheres to our policy

Input files (fasta and genbank) for the GWAS analysis are available on figshare (doi:10.6084/M9.FIGSHARE.20526837.V1, doi:10.6084/M9.FIGSHARE.20526891.V1, doi.org/10.6084/m9.figshare.11879340.v1). Other inputs (unitigs, gene presence/absence, phylogenetic tree) used to run the GWAS are also available on figshare (doi:10.6084/M9.FIGSHARE.20526852.V1, doi:10.6084/M9.FIGSHARE.20526858.V1, doi:10.6084/M9.FIGSHARE.20526885.V1).
Output from the CC87 GWAS analysis are available on figshare (doi:10.6084/M9.FIGSHARE.20526855.V1, doi:10.6084/M9.FIGSHARE.20526861.V1).
Ppanggolin HDF5 output file contains all results from ppanggolin and panRGP analysis and is available on figshare (doi:10.6084/M9.FIGSHARE.21435816.V1).
The bioprojects and accession numbers of the CC87 genomes are available in Supplementary Data 1. The assemblies and annotation of the 370 strains of Escherichia are available on figshare (https://doi.org/10.6084/m9.figshare.11879340.v1, https://doi.org/10.6084/m9.figshare.19536163.v1). The 2302 complete genomes of E. coli can be obtained from RefSeq (https://www.ncbi.nlm.nih.gov/assembly) using the accession numbers available in Supplementary Data 11. Source data are provided with this paper.
Three fully sequenced reference genomes of E. coli were used to map back significant unitigs: CVM_N16EC0879 (GenBank: CP043744.1, https://www.ncbi.nlm.nih.gov/assembly/GCA_008386415.1/), IAI1 (Refseq: NC_011741.1, https://www.ncbi.nlm.nih.gov/assembly/GCF_000026265.1/), S88 (Genbank: CU928161.2, https://www.ncbi.nlm.nih.gov/assembly/GCA_000026285.1)

Human research participants

Policy information about studies involving human research participants and Sex and Gender in Research.

Reporting on sex and gender	NA
Population characteristics	NA
Recruitment	NA
Ethics oversight	NA

Note that full information on the approval of the study protocol must also be provided in the manuscript.

Field-specific reporting

Please select the one below that is the best fit for your research. If you are not sure, read the appropriate sections before making your selection.

☐ Life sciences ☐ Behavioural & social sciences ☒ Ecological, evolutionary & environmental sciences

For a reference copy of the document with all sections, see nature.com/documents/nr-reporting-summary-flat.pdf

Ecological, evolutionary & environmental sciences study design

All studies must disclose on these points even when the disclosure is negative.

Study description	The study aims to decipher the genetic determinants involved in virulence both at the clonal (CC87) and species level. To this end, it used a mouse model of sepsis coupled with a genome-wide association study to identify the most relevant genes. Particular emphasis is placed on genes related to iron acquisition in terms of prevalence, co-occurrence and genomic location (i.e. chromosome or plasmid).
Research sample	A first dataset consists of 232 strains belonging to the clonal complex 87 (CC87). This dataset gathered strains and genomes from several origins (described in Supplementary Data 1) as well as genomes obtained from a previous study by Reid et al. (PMID: 35115531). This dataset has been used because it is representative of the whole diversity of the CC87, including both ST58 and ST155 strains.<br>The second dataset is composed 370 genomes representative of the genus Escherichia and which has been previously described (PMID: 33112851).

	Finally, the third dataset consists of the complete E. coli genomes available on RefSeq on September 19, 2022. This dataset has been used because it is composed of high-quality circularized genomes (chromosome +/- plasmids) from strains belonging to the main the E. coli phylogroups.
Sampling strategy	No sample size calculation was performed. The genomes from the first dataset (232 CC87 genomes) were obtained from various strains as described in Supplementary Data 1. These strains were chosen because they were diverse in terms of sequence type (ST58 or ST155), source, host and pathotype. The genomes from the second dataset (370 Escherichia genomes) were chosen because they are representative of the whole genus diversity (E. fergusonii. E. albertii, Escherichia clades, main E. coli phylogroups). The genomes from the third dataset (2302 complete genomes of E. coli) were chosen because they consist of all available complete genome of E. coli on RefSeq on September 19, 2022.
Data collection	Among the CC87 dataset, 206 genomes and metadata were obtained from strains collected through different studies (described in Supplementary Data 1) and 26 genomes and metadata were retrieved from the study by Reid et al. (PMID: 35115531). The 370 genomes of Escherichia were obtained from figshare (https://doi.org/10.6084/m9.figshare.11879340.v1, https://doi.org/10.6084/m9.figshare.19536163.v1). The 2302 complete genomes of E. coli were obtained from RefSeq (https://www.ncbi.nlm.nih.gov/assembly).
Timing and spatial scale	The strains from the first dataset were obtained from France, French Guyana, Madagascar, Australia, Senegal, Mali, USA and Asia. Sampling dates range from 1980 to 2020. The timing and spatial scale was chosen to compile genome from diverse geographical origin and time of sampling. Full information is avalaible in Supplementary Data 1.<br>For the strains of the second (370 Escherichia genomes) and third (2302 complete E. coli genomes) data sets, we had no specific criteria in terms of timing or spatial sampling.
Data exclusions	No data were excluded from the analysis.
Reproducibility	Mouse sepsis assay were done 10 times for each tested strains. Negative and positive controls were included in each experiment. In all cases, we obtained similar results for the controls, mice being killed only by CFT073 at the same time after the inoculation +/- 4h.
Randomization	The genomes from the CC87 were groupes based on their phylogenetic history in congruence with sequence types.<br>The complete genomes from RefSeq were grouped based on phylogroups and sequence types.
Blinding	The mice were inoculated in a blind experiment by the zootechnician that ignores the status of the strain.

Did the study involve field work? ☐ Yes ☒ No

Reporting for specific materials, systems and methods

We require information from authors about some types of materials, experimental systems and methods used in many studies. Here, indicate whether each material, system or method listed is relevant to your study. If you are not sure if a list item applies to your research, read the appropriate section before selecting a response.

Materials & experimental systems

n/a	Involved in the study
☒	☐ Antibodies
☒	☐ Eukaryotic cell lines
☒	☐ Palaeontology and archaeology
☐	☒ Animals and other organisms
☒	☐ Clinical data
☒	☐ Dual use research of concern

Methods

n/a	Involved in the study
☒	☐ ChIP-seq
☒	☐ Flow cytometry
☒	☐ MRI-based neuroimaging

Animals and other research organisms

Policy information about studies involving animals; ARRIVE guidelines recommended for reporting animal research, and Sex and Gender in Research

Laboratory animals	Female mice OF1 of 14–16 g (4 week-old) from Charles River (L'Arbresle, France) were used for the mouse sepsis assay. Housing conditions for the mice were in agreement with the French law, with dark/light cycle, and constant ambient temperature (21°C +/- 2°C) and humidity (50% +/- 10%).
Wild animals	The study did not involve wild animals
Reporting on sex	Only female mice were used.
Field-collected samples	The study did not involve animals collected from the fiels.

Ethics oversight	The protocol (APAFIS#4948) was approved by the French Ministry of Research and by the Ethical Committee for Animal Experiments, CEEA-121, Comité d'éthique Paris-Nord.

Note that full information on the approval of the study protocol must also be provided in the manuscript.
